# Observation of changes after peripheral retinal injury by cosmetic laser, using wide-field scanning laser ophthalmoscope: A case report

**DOI:** 10.1097/MD.0000000000014354

**Published:** 2019-02-08

**Authors:** Rae Young Kim, Ho Ra

**Affiliations:** Department of Ophthalmology and Visual Science, College of Medicine, The Catholic University of Korea, Seoul, Korea.

**Keywords:** cosmetic laser, laser injury, wide-field scanning laser ophthalmoscopy

## Abstract

**Rationale:**

Increases in cosmetic laser use have led to recent reports of accidental retinal injuries, most of which are limited to the posterior pole. We report a case of peripheral retinal injury caused by a 1064-nm Nd:YAG: neodymium-doped yttrium aluminum garnet.

**Patient concerns:**

A 27-year-old Asian woman was admitted with scotoma symptoms in her right eye. The patient was a skin care technician. Three days before admission, a laser beam had struck her eye while she was preparing for a laser procedure.

**Diagnosis:**

During fundus examination, a subretinal hemorrhage with disc diameter (DD) of 4.0 and a preretinal hemorrhage of 2.5 DD in its center were found in the 2 o’clock position of the peripheral retina in the right eye.

**Interventions:**

We monitored the injury for > 6 months, first using fluorescein angiography, then wide-field scanning laser ophthalmoscopy and optical coherence tomography. Oral steroids and vitamins were administered.

**Outcomes:**

During the 6-month follow-up period, blood from the initial sub- and preretinal hemorrhage, as well as vitreous hemorrhage, were all absorbed. Retinal detachment was not observed as scar formation and adhesions had occurred. No interventions were considered necessary.

**Lessons:**

When treating a patient who has experienced laser eye injury, the possibility of peripheral retinal injury should be considered. Peripheral retinal injury caused by 1064-nm Nd:YAG: neodymium-doped yttrium aluminum garnet has a relatively good prognosis, suggesting that it will not progress to retinal detachment.

## 1 Introduction

Laser instruments are used in many spheres of human activity, including medicine, industry, laboratory research, entertainment, and the military. This widespread use of lasers has resulted in many accidental injuries,^[1]^ with the eyes experiencing the highest percentage of these.^[2]^ Within the eye, the retina is known to be especially vulnerable; as the laser beam is fired in a relative short period of time from a certain distance, injuries occur mostly in the retinal posterior pole of a single eye.^[1]^ There have been recent reports regarding various retinal complications caused by cosmetic laser devices, most of which are limited to the retinal posterior pole.^[3–8]^ Chen et al ^[9]^ reported a peripheral retinal injury that occurred 5 disc diameters (DD) below the macula; however, they limited their report to progress monitoring for 3 days following the injury, because of patient loss. We report on a case involving peripheral retinal injury caused by 1064-nm Nd:YAG: neodymium-doped yttrium aluminum garnet. We monitored the injury for > 6 months using wide-field scanning laser ophthalmoscopy.

## 2 Case presentation

The Institutional Review Board/Ethics Committee of Bucheon St. Mary's Hospital approved this study. It was performed in accordance with the tenets of the Declaration of Helsinki. Written informed consent was obtained from the patient for publication of this case report and any accompanying images.

A 27-year-old Asian woman was admitted for symptoms of scotoma in her right eye in late August 2016. The patient was a skin care technician. Three days prior to the admission, a laser beam had struck her eye while she was preparing for a laser procedure; immediately after sustaining the injury, she developed symptoms of scotoma in the inferotemporal area of the right eye. The laser device used at the time was a 1064 /532 nm Nd:YAG cosmetic laser (Spectra VRM IV, Lutronic Corporation, Goyang City, South Korea). While preparing for the laser treatment, the device held in her hand accidentally fired a laser beam from a distance of approximately 20 cm in a position to the right and below the eye, which injured her right eye. The patient was not wearing protective goggles or eye glasses at the time. She did not recall the exact power or duration of the laser used.

Her visual acuity, measured by the Snellen chart at our hospital, was 20/20 in both right and left eyes, while the intraocular pressures were 16 and 17 mm Hg in the right and left eyes, respectively; thus, all results were within the normal range. In the light reflex test, the patient showed normal responses in both eyes, while she also showed no signs of relative afferent pupillary defect (RAPD).

**Figure 1:**
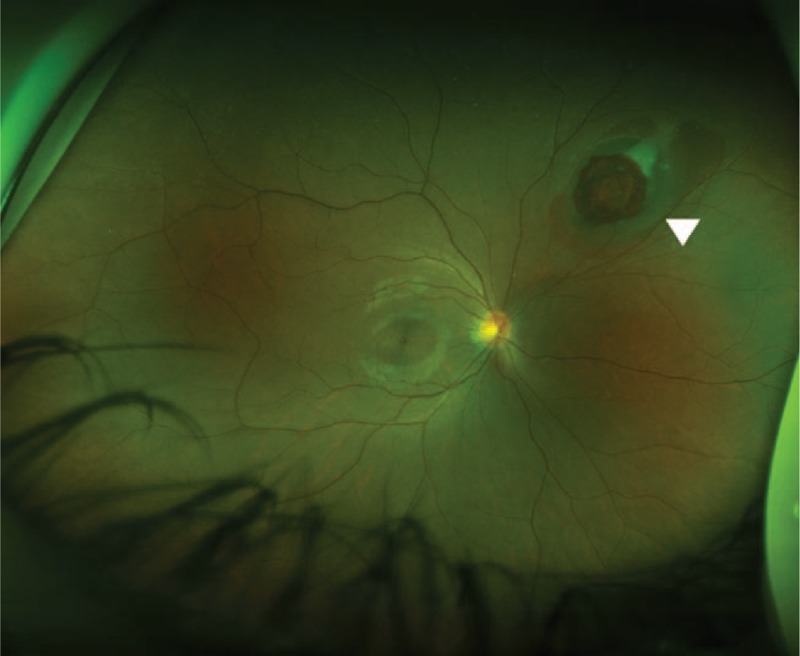
Wide-field scanning laser ophthalmoscopy image at 3 days after the injury. The 4.0 DD subretinal hemorrhage with a 2.5 DD preretinal hemorrhage in its center was found in the 2-o’clock direction of the peripheral retina in the right eye.

**Figure 2:**
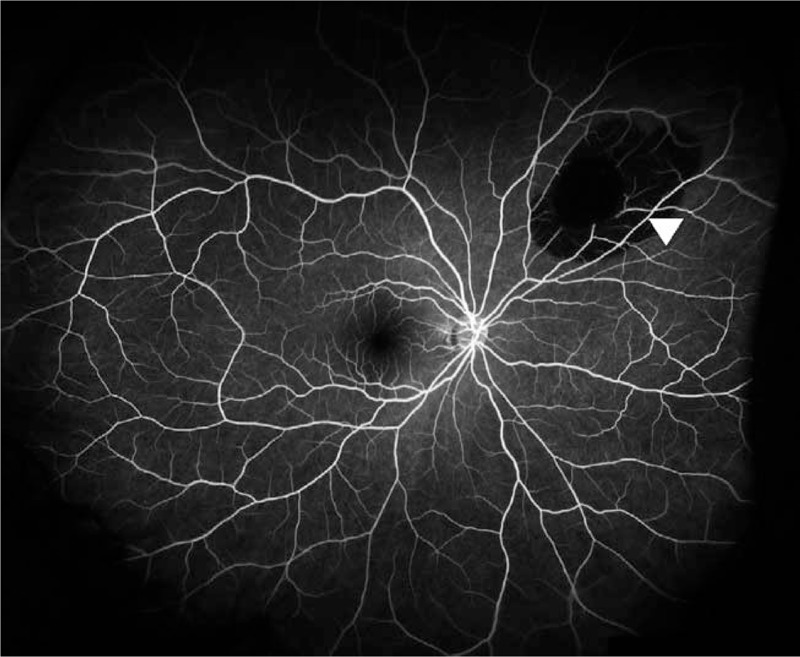
Fluorescein angiography image at 3 days after the injury. Hypofluorescence due to blockage caused by the pre- and subretinal hemorrhage was found in the lesion area.

In the slit-lamp anterior segment examination, neither eye showed any findings of anterior chamber inflammation or lens opacity. In the wide-field scanning laser ophthalmoscopy and fundus examinations, after 3 rounds of pupillary dilation in 10-minute increments using Mydrin-P, we discovered a 4.0 DD subretinal hemorrhage with a 2.5 DD preretinal hemorrhage in its center in the 2-o’clock direction of the peripheral retina in the right eye (Fig. 1). Subsequently, fluorescein angiography was performed after a skin hypersensitivity test and showed hypofluorescence due to blockage caused by the pre- and subretinal hemorrhages (Fig. 2).

As the patient showed no symptoms of vision loss other than peripheral scotoma, nor was there peripheral retinal detachment, the treatment plan was to administer 15 mg of oral steroids once a day for 2 weeks before tapering it, and vitamins for 3 months while monitoring her progress.

**Figure 3:**
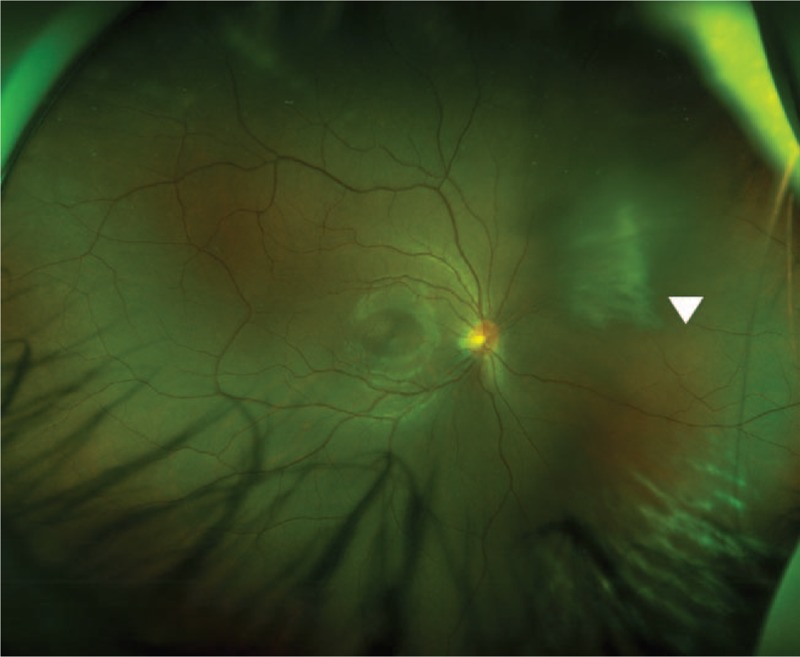
Wide-field scanning laser ophthalmoscopy image at 2 weeks after the injury. Vitreous hemorrhage and opacity were found in front of the lesion.

**Figure 4:**
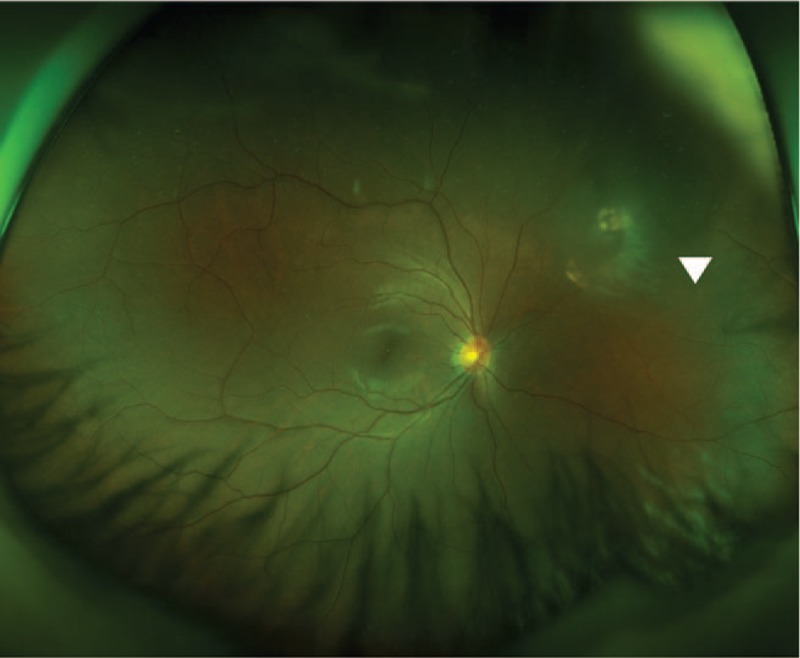
Wide-field scanning laser ophthalmoscopy image at 1 month after the injury. Decrease in vitreous hemorrhage and opacity were observed, along with an atrophic scar and localized fibrosis in the lesion area.

During the follow-up at 1 week after the injury, the subretinal hemorrhage in the lesion did not show significant change in size, while the preretinal hemorrhage decreased in size. In the follow-up at 2 weeks after the injury, vitreous opacity and hemorrhage were both found in front of the lesion (Fig. 3). At 1 month after the injury, vitreous opacity and hemorrhage had decreased, and the patient showed signs of recovery with an atrophic scar and localized fibrosis in the lesion area; notably, she did not exhibit any signs of retinal detachment (Fig. 4).

**Figure 5:**
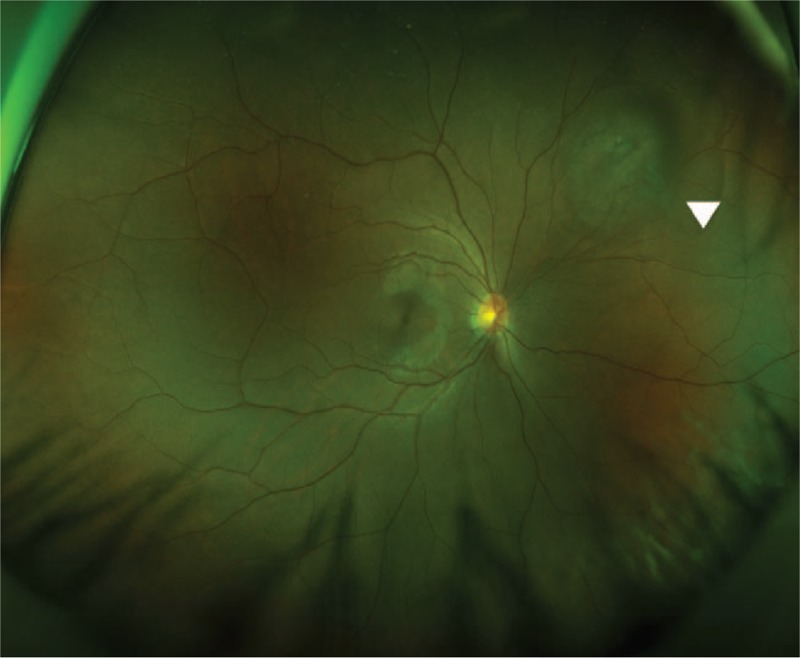
Wide-field scanning laser ophthalmoscopy image at 6 months after the injury. Vitreous opacity decreased and there were no signs of retinal tear or detachment.

**Figure 6:**
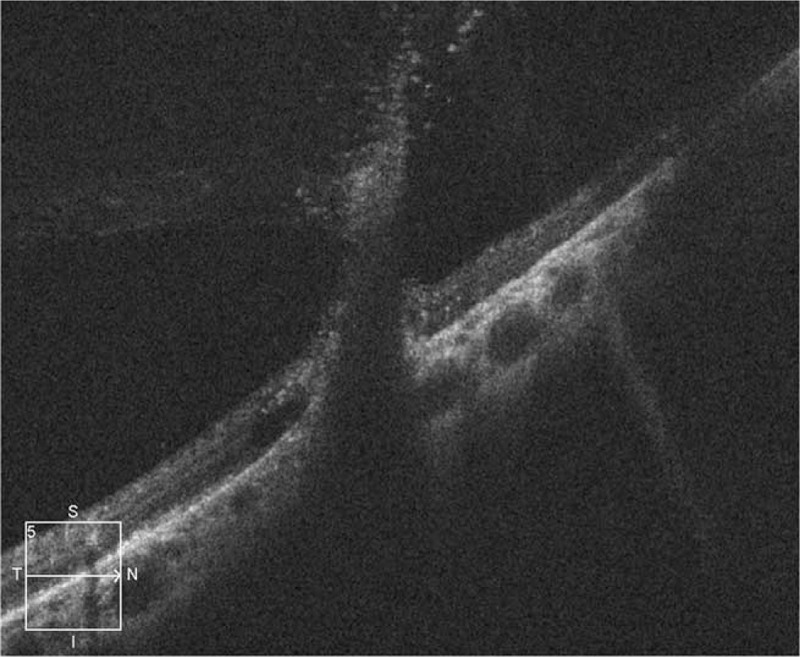
Optical coherence tomography examination at 6 months after the injury, showing vitreous traction.

At 4 months after the injury, the blood from both the sub- and preretinal hemorrhages had been absorbed, but mild vitreous opacity remained. The patient continued to complain of peripheral scotoma. At 6 months after the injury, vitreous opacity decreased and there were no signs of retinal tear or detachment (Fig. 5). However, the patient's scotoma symptoms remained. Optical coherence tomography (OCT) examination of the lesion area at 6 months after injury showed evidence of vitreous traction (Fig. 6). We planned a further 3 months of outpatient follow-up.

## 3 Discussion and Conclusions

Recent increase in the use of cosmetic laser devices in various fields has resulted in increased reports of retinal complications.^[3–8]^ Asiri et al^[4]^ reported 3 cases of retinal posterior pole injuries caused by alexandrite lasers, with macular choroidal neovascular membrane occurring as a possible secondary complication. Shum et al^[3]^ reported on retinal injury in the periphery of the optic disc, caused by 1064 nm Nd:YAG cosmetic laser, where 6 months of follow-up observations did not detect retinal detachment. However, scotoma persisted in the lesion area as scar formation occurred after the hemorrhagic blood was absorbed. Qi et al^[8]^ reported that 11 patients with laser-induced macular holes showed favorable surgical outcomes. In the aforementioned cases, retinal complications caused by cosmetic laser occurred mostly in the retinal posterior pole.^[1]^ Most cosmetic laser accidents occur in indoor settings, where the laser beam is nondispersed because of the close proximity to the laser source; this nondispersed laser beam passes through a narrow pupil to cause injury to the retina. Consequently, mostly injuries occur in the posterior pole of a single eye. However, in cases where the laser beam incident angle is unique, as in our case, injury may also occur in the peripheral retina. The single most important factor in determining the degree of functional ocular damage is the retinal location of the laser injury. Thus, injuries that are closer to the fovea are known to have poorer prognoses.^[10]^ However, even in cases of peripheral retinal injury, it should be noted that initial vision loss may occur from retinal or vitreous hemorrhage, and late-onset vision loss due to retinal detachment caused by a ruptured retina may also occur.

Similar to our case, Chen et al^[9]^ reported a peripheral retinal injury that occurred approximately 5 DD below the posterior pole; however, they were only able to report monitoring for 3 days following the injury because of patient loss. As our case was similar to that of Chen et al,^[9]^ we considered changes in hemorrhaging over time and possible onset of retinal detachment. During the 6-month follow-up period, the blood from initial sub- and preretinal hemorrhage was absorbed; moreover, retinal detachment did not occur as scar formation took place, similar to the case reported by Shum et al.^[3]^

Laser-tissue interaction can be classified as photocoagulative, photodisruptive, and photoablative.^[3]^ In general, visible wavelengths produce photocoagulation, ultraviolet wavelengths produce photoablation, and infrared can produce either photodisruption or photocoagulation.^[3]^ Injuries caused by 1064-nm Nd:YAG: neodymium-doped yttrium aluminum garnet, which was used in our study, may be viewed as involving laser-induced photodisruption. We suspect that, similar to the histologic study by Manning et al,^[10]^ the lesion in our case involved disruption of all retinal layers, a break in Bruch membrane, and choroidal vessel rupture to cause subretinal hemorrhage, which eventually leaked to the preretina to form preretinal hemorrhage. As the blood from the hemorrhage was absorbed, scar formation from fibrosis occurred and resulted in adhesion rather than progression into retinal detachment.

To our knowledge, our case report is the first with at least 6 months of follow-up by wide-field scanning laser ophthalmoscopy of a peripheral retinal injury caused by a cosmetic laser. Although this is only a single case and a larger number of cases are required to draw definitive conclusions, peripheral retinal injury caused by 1064-nm Nd:YAG: neodymium-doped yttrium aluminum garnet appears to exhibit a relatively good prognosis and may not progress to retinal detachment.

## Author contributions

**Conceptualization:** Ho Ra.

**Data curation:** Rae Young Kim.

**Investigation:** Ho Ra.

**Writing – original draft:** Rae Young Kim.

**Writing – review & editing:** Ho Ra.
